# Wnt signaling in lung development, regeneration, and disease progression

**DOI:** 10.1038/s42003-021-02118-w

**Published:** 2021-05-20

**Authors:** Cody J. Aros, Carla J. Pantoja, Brigitte N. Gomperts

**Affiliations:** 1 UCLA Department of Molecular Biology Interdepartmental Program, UCLA, Los Angeles, CA USA; 2 UCLA Medical Scientist Training Program, David Geffen School of Medicine, UCLA, Los Angeles, CA USA; 3 UCLA Children’s Discovery and Innovation Institute, Mattel Children’s Hospital UCLA, Department of Pediatrics, David Geffen School of Medicine, UCLA, Los Angeles, CA USA; 4 Division of Pulmonary and Critical Care MedicineDavid Geffen School of Medicine, UCLA, Los Angeles, CA USA; 5 Jonsson Comprehensive Cancer Center, UCLA, Los Angeles, CA USA; 6 Eli and Edythe Broad Stem Cell Research Center, UCLA, Los Angeles, CA USA

**Keywords:** Cell signalling, Lung cancer, Developmental biology, Respiration

## Abstract

The respiratory tract is a vital, intricate system for several important biological processes including mucociliary clearance, airway conductance, and gas exchange. The Wnt signaling pathway plays several crucial and indispensable roles across lung biology in multiple contexts. This review highlights the progress made in characterizing the role of Wnt signaling across several disciplines in lung biology, including development, homeostasis, regeneration following injury, in vitro directed differentiation efforts, and disease progression. We further note uncharted directions in the field that may illuminate important biology. The discoveries made collectively advance our understanding of Wnt signaling in lung biology and have the potential to inform therapeutic advancements for lung diseases.

## Abstract (web summary)

Cody Aros, Carla Pantoja, and Brigitte Gomperts review the key role of Wnt signaling in all aspects of lung development, repair, and disease progression. They provide an overview of recent research findings and highlight where research is needed to further elucidate mechanisms of action, with the aim of improving disease treatments.

## Introduction

The lung is a structurally and functionally intricate organ, with over 40 different, known cell types^1^. The advent of single-cell sequencing and other technologies continues to increase this number and contributes to the striking complexity of the lung. At its most proximal portion, the cartilaginous conducting airways harbor a pseudostratified mucociliary epithelium that plays a vital role in host defense. Inhaled harmful particles, pathogenic organisms, and debris are expectorated from the airways via a process known as mucociliary clearance (MCC). Inhaled contents are first entrapped by a layer of mucus, produced by goblet cells, that are then transported proximally by the unidirectional beating of cilia from terminal bronchioles to the trachea^2^. The coordinated removal of debris by ciliated cells and mucus-producing goblet cells is facilitated by lubrication from a periciliary water layer. The concerted undertaken by these multiple cell types together comprise the mucociliary escalator^2^. The bronchioles, also referred to as the conducting airways, are additionally important for moving gases to and from the distal lung, which contain the alveolar sacs necessary for gas exchange. Atmospheric oxygen undergoes exchange for blood carbon dioxide, a process that promotes cellular respiration for all tissues of the body.

**Fig. 1:**
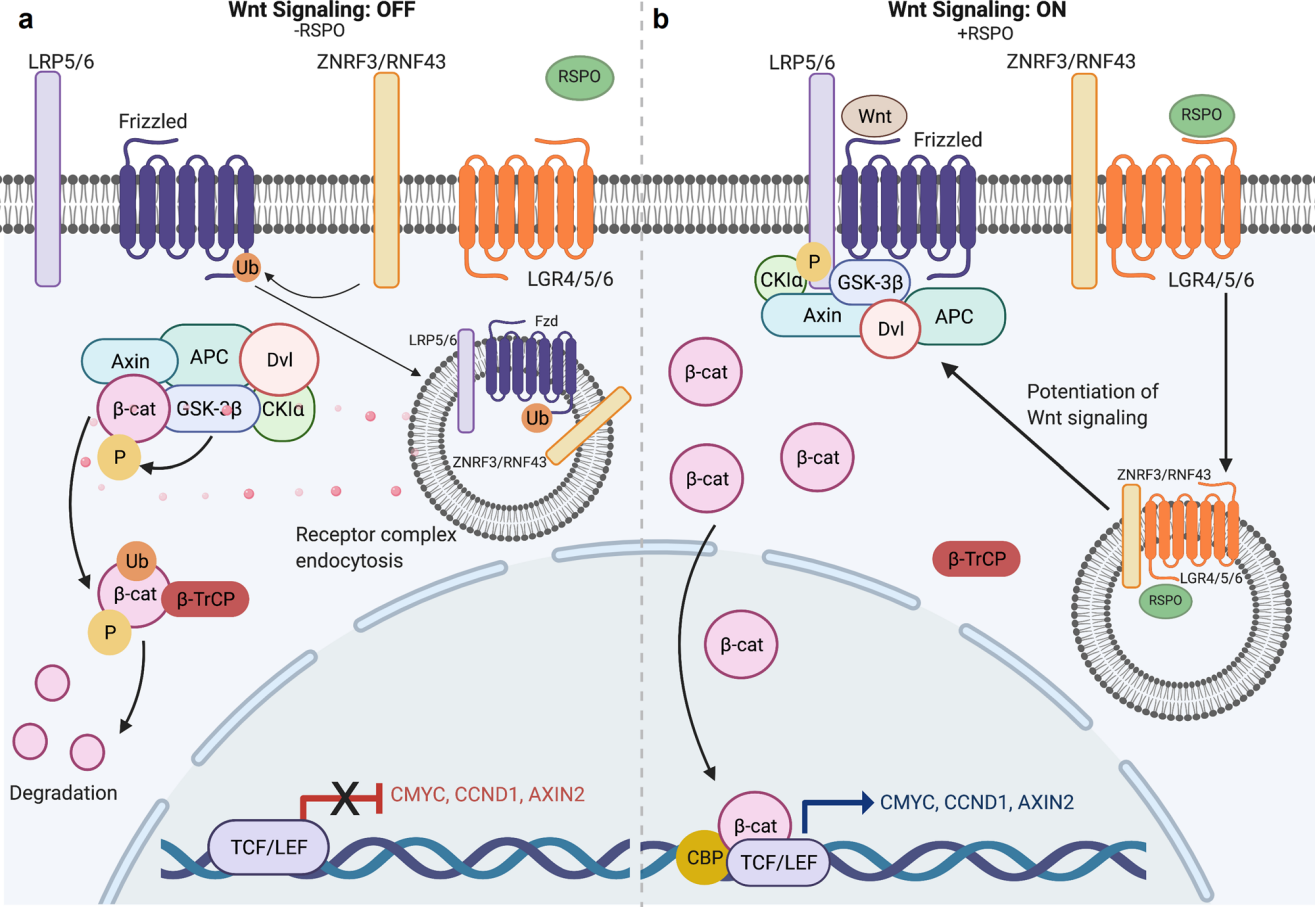
Overview of canonical Wnt signaling. **a** In the absence of RSPO binding to LGR4/5/6, ubiquitin ligase ZNRF43/RNF43 ubiquitinates the Frizzled receptor which leads to receptor complex endocytosis, β-catenin degradation and subsequent inhibition of Wnt-driven transcriptional activity^5^. **b** The binding of RSPO to LGR4/5/6 potentiates Wnt signaling by removing ZNR43/RNF43 ubiquitin ligase from the cell membrane, which would otherwise mark Frizzled receptor for ubiquitination. Frizzled receptors are then able to interact with both Wnt ligand and LRP5/6 co-receptor to drive Wnt signaling cascade. β-catenin then escapes cytoplasmic proteasomal degradation, resulting in its nuclear translocation, interactions with transcription factors. TCF/LEF, and subsequent transactivation of Wnt target genes like c-MYC, CyclinD1, and Axin2^5^.

The wingless related-integration site (Wnt)/β-catenin signaling pathway plays an instrumental role in stem cell self-renewal across several tissue epithelia^3^. R-spondin ligands are cysteine-rich glycoproteins that bind to their cognate leucine-rich repeat-containing G-protein coupled receptor (LGR) LGR4/5/6 receptors and E3 ubiquitin ligases ring finger protein (RNF)43/ZNRF3 via their furin-like domains^4,5^. In the absence of R-spondins, RNF43/ZNR43 ubiquitinate the Frizzled receptors that targets them for degradation (Fig. 1a, b). As such, Wnt signaling is dampened. Under canonical conditions in the absence of Wnt ligand, β-catenin is in complex with several other proteins including Adenomatous polyposis coli (APC), Glycogen synthase kinase-3a, Glycogen synthase kinase-3β (GSK3β), casein kinase I (CKI), Axin, and Disheveled, among others (Fig. 1a)^3^. These proteins together comprise a destruction complex. CKIγ first phosphorylates β-catenin at residue serine 45, a priming event that allows for recognition by GSK3β, which phosphorylates β-catenin at serine 33, serine 37, and threonine 41^3^. These N-terminal post-translational modifications mark β-catenin for ubiquitination and subsequent proteasomal degradation by the E3 ubiquitin ligase β-transducin repeat-containing protein (Fig. 1a).

However, upon Wnt transcription and translation, the ligand enters to the endoplasmic reticulum (ER) and encounters Porcupine, an ER-resident protein that palmitoylates N-terminal cysteine residues of Wnt proteins. This lipid tail modification has been shown to be necessary for Wnt ligand secretion^6,7^. Wnt ligand then binds to LRP5/6 and appropriate Frizzled co-receptor on the same or neighboring cell. Moreover, R-spondins can potentiate and amplify Wnt signaling by forming complexes with their cognate LGR pair and binding to RNF43/ZNR43 to prevent Frizzled receptor ubiquitination. Together, these events allow for a cascade of molecular events that results in disassembly of the cytoplasmic destruction complex (Fig. 1b). β-catenin then accumulates in the cytoplasm, subsequently translocates to the nucleus, and interacts with transcription factors T-cell factor/Lymphoid enhancer factor 1 (TCF/LEF) (Fig. 1b). In this way, β-catenin drives transactivation of downstream target genes such as c-MYC, AXIN2, and CYCLIN D1 among others (Fig. 1b). The canonical Wnt signaling described above is important in a variety of cellular processes including proliferation, self-renewal, epithelial to mesenchymal transitions, and migration and motility.

In contrast, non-canonical Wnt signaling is often thought of as the β-catenin-independent pathway. One arm of this pathway regulates planar cell polarity (PCP), during which Frizzled receptors trigger downstream activation of RhoA and Rac GTPases that promote cytoskeletal remodeling^8^. A second arm of non-canonical Wnt signaling lies in Wnt-Frizzled binding that triggers Phospholipase C and downstream Ca^2+^ activity for regulation of cell migration and fate decisions^8^. It is important to note that Porcupine palmitoylates all 19 mammalian Wnt ligands and is therefore necessary for their secretion, including those that partake in non-canonical signaling.

Over the past several years, much research has been put forth toward carefully dissecting the nuanced role of Wnt signaling across several disciplines pertaining to lung biology. This review aims to highlight the major contributions made to our current understanding of the Wnt signaling pathway in lung and airway development, its role in proximal and distal airway homeostasis and relevant niche biology, as well as its role in directed differentiation efforts of induced pluripotent stem cells (iPSCs) and embryonic stem cells (ESCs) to the lung lineage, in organoid culture models, and its perturbations in disease states.

## Development

Detailed staging and mechanisms of both human and murine lung development have been well reviewed by others^9–12^. To briefly summarize murine lung development, during the embryonic stage, the ventral anterior foregut endoderm (AFE) expresses the transcription factor Nkx2.1 at mouse embryonic day (E) 9.0 as a sign of the specification to promote initial lung budding. From E9.5–E12.5, two lung buds with high Nkx2.1 expression and a proximal portion with low Nkx2.1 that later forms the trachea emerge concomitantly with tracheo-esophageal septation. From E12.5–E16.5 during the pseudoglandular stage, the lung buds undergo a period of branching morphogenesis to form the lung tree and terminal bronchioles. Upon completion of the canalicular and saccular stages of development (E16.5-Postnatal (P) day 4), the terminal bronchioles narrow and begin to form epithelial sacs. These structures later form fully mature alveolar structures for gas exchange by P21 during the alveolarization phase^9^. In contrast, alveolarization begins pre-partum during human lung development and continues postnatally into childhood^13^.

Coordinated development of the conducting and distal airways is a vital process during which both the epithelial and mesenchymal compartments play integral roles. The developing lung endodermal buds penetrate the splanchnic mesoderm and mesothelium around E9.5. The developing distal lung then acquires four distinct layers, each with its own unique anatomical, cellular, morphologic, and molecular profiles: endoderm (epithelium), subepithelial mesoderm (mesenchyme), submesothelial mesoderm (mesenchyme), and mesothelium. Transient amplifying submesothelial cells give rise to a parabronchial smooth muscle cell (PSMC) progenitor population. This cell population then migrates more proximally around the bronchi and differentiates into smooth muscle cells (SMCs)^14,15^. Studies assessing the expression levels of various Wnt/β-catenin signaling members and activity reporters in these aforementioned compartments during lung development have identified contrasting findings^16,17^. However, a myriad of studies collectively demonstrates that the developing lung mesenchyme displays several highly regulated interactions with the lung endoderm in both mouse and human that together coordinate normal lung organogenesis^9^, many of which are Wnt-mediated.

### Embryonic stage (E9.5–E12.5)

Wnt signaling plays a role in some of the earliest stages of cardiopulmonary specification. Wnt2+ Gli1+ Isl1+ cells comprise the multipotent cardiopulmonary mesoderm progenitors (CPPs) that orchestrate heart and lung development. Lineage tracing of Wnt2+ CPPs at E8.5 demonstrates their capacity to generate the cardiac inflow tract and pulmonary mesoderm cell lineage by E17.5^18^. These cells are important for the vital epithelial–mesenchymal interactions that occur during lung development.

### Crosstalk from developing mesenchyme to endoderm

**Fig. 2:**
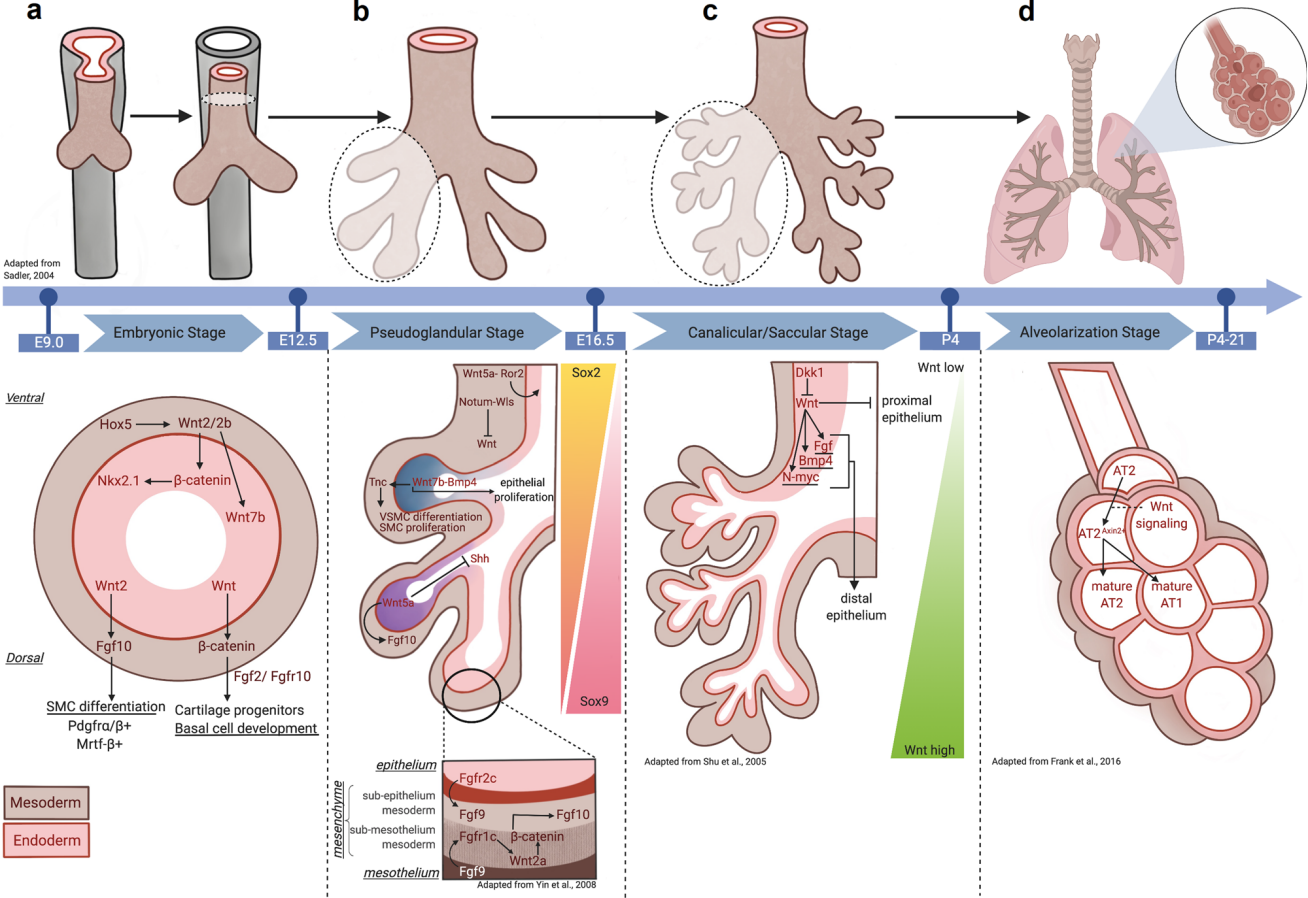
Wnt signals mediate epithelial–mesenchymal interactions during lung development. **a** During the embryonic stage (E9.0–12.5) of development, lung bud emerges from tracheal-esophageal septation occurs^9^. Mesenchymal (brown) HOX5 spatiotemporally regulates endodermal (pink) Wnt2/2b to establish NKX2.1 lung progenitor via downstream β-catenin signaling^21–24^. Endodermal Wnt ligands also promote mesenchymal FGF10 and β-catenin, which then allow for SMC differentiation and cartilage and basal cell development^27^. **b** During the pseudoglandular stage (E12.5–E16.5), lung buds undergo branching morphogenesis to develop terminal bronchioles^9^. Mesenchymal Wnt5a promotes tracheal and cartilage formation via ROR2-dependent mechanisms^33^. Wntless (Wls)-regulated Notum suppresses mesenchymal Wnt and is necessary for tracheal development and branching morphogenesis^39,40^. A Wnt7b-BMP4 signaling axis also promotes epithelial proliferation and mesenchymal vascular SMC (VSMC) differentiation and SMC proliferation^35–38^. Further, epithelial Wnt5a expression is highest in distal tips^31,33^ and works to promote branching morphogenesis via suppression and activation of SHH and Fgf10 signaling, respectively^34^. A closer examination of the mesenchyme reveals FGF9 signaling from both the epithelium and mesothelium converge to promote submesothelial Wnt2a and facilitate mesenchymal cell proliferation^14^. **c** During the canalicular/saccular stages of development (E16.5-P4), terminal bronchioles become more defined and form epithelial sacs^9^. Negative regulation of Wnt by DKK1 results in proximalization of lung epithelium. High levels of Wnt signaling drive a distal airway phenotype, mediated in part by N-MYC-BMP4-FGF signaling^44^. **d** The alveolarization stage (P4–21) concludes with the maturation of alveolar structures^13^. Wnt-responsive (AXIN2+) ATII cells regulate lung alveologenesis by skewing toward a mature ATII lineage and in lieu of an ATI lineage^47^.

Wnt-driven mesenchymal-to-endodermal crosstalk is critically important from the earliest stages of lung development. From E9.5–12.5, there is active Wnt/β-catenin signaling in both the epithelium and the mesenchyme adjacent to the future proximal airway as measured by TOPGAL and AXIN2-LacZ Wnt activity reporters^19,20^. Canonical Wnt2/2b ligands are spatiotemporally regulated by Hox5 genes during this stage, with notable mesodermal expression near the ventral aspect of the anterior foregut between E9.0 and E10.5^21^ (Fig. 2a). Together, Wnt2/2b cooperate to promote Nkx2.1+ lung endodermal specification (Fig. 2a), as mice without them display lung agenesis^22–24^. Wnt2/2b converges on canonical signaling in the developing endoderm, as deletion of β-catenin in the anterior foregut also display lung agenesis, resulting in the upregulation of a Sox2+ digestive progenitor identity^19,22^. In contrast, constitutive activation of β-catenin prevents tracheo-esophageal septation and instead drives Nkx2.1+ lung endodermal progenitor expansion^19,22^.

The developing embryonic lung also has measures in place to curb canonical Wnt signaling. While homeobox protein 1 (Barx1) is prominently expressed in the developing stomach mesenchyme where it orchestrates endodermal differentiation, its expression throughout the dorsal foregut mesenchyme and mainstem bronchi has been shown to inhibit endodermal Wnt signaling to promote thoracic foregut specification^25^. Similar to constitutive β-catenin activation, deletion of Barx1 results in the loss of tracheo-esophageal septation as evidenced by single, contiguous luminal layer of Nkx2.1+ tracheal and Sox2+ esophageal epithelium by E10.5^19,22,25^.

### Crosstalk from developing endoderm to mesenchyme

As early as E11.5, canonical Wnt2 ligand signaling promotes fibroblast growth factor 10 (Fgf10) signaling that, in turn, facilitates differentiation of immature platelet-derived growth factor receptor (Pdgfr)α/β+ smooth muscle cells by regulating the expression of myocardin and Mrtf-B, critical transcription factors in myogenesis^26^ (Fig. 2a). Mesenchymal Wnt2 also promotes endodermal Wnt7b expression that later signals back to the subepithelial mesenchyme to further drive SMC differentiation^26^. More recently, epithelial Wnt ligands were shown to activate mesenchymal β-catenin, which alongside Fgf10/Fgfr2, regulate both cartilage progenitors and basal cell development^27^ (Fig. 2a).

### Pseudoglandular stage (E12.5–E16.5)

Constitutive activation of β-catenin in surfactant protein C (Sftpc)+ cells drives distal conducting airway dilatation concomitant with no differentiation to either the secretory or ciliated cell fate^28^. Consistent with this, R-spondin 2 (Rspo2) facilitates normal embryonic lung growth and the start of branching morphogenesis by potentiating Wnt/β-catenin signaling^29^. A recent study identified, however, that Rspo2 antagonizes RNF43 and zinc and ring finger (ZNRF)3 to regulate limb and lung formation in xenopus^4^. These efforts resulted in a paradigm shift, demonstrating that Rspo2 can potentiate Wnt signaling in the absence of LGR^4,30^. Interestingly, while Rspo2 drives branching morphogenesis earlier in development, it has no role in the differentiation of epithelial or mesenchymal cell types^29^. This suggests functional redundancy in the varying upstream regulators of Wnt signaling in embryonic lung development.

Several Wnt ligands have been well characterized in the pseudoglandular stage. Wnt5a is diffusely expressed in both the epithelium and mesenchyme as early as E12.0^31^ (Fig. 2b). Its expression is dynamic and highest in the distal epithelium at branching point tips at E16.0 and is critical for tracheal elongation, cartilage ring patterning, and constraining distal airway expansion^31–33^. Furthermore, epithelial Wnt5a regulates distal lung morphogenesis via suppression and activation of sonic hedgehog (Shh) and Fgf10 signaling, respectively^34^. Mesenchymal Wnt5a is primarily responsible for tracheal elongation and cartilage ring formation via Ror2-dependent mechanisms in the dorsal tracheal mesenchyme^33^.

Wnt2a is expressed in the distal lung submesothelial layer during branching morphogenesis^20^. Epithelial- or mesothelial-derived FGF9 triggers activation of mesenchymal FGFR1/2 at E13.5^14^. Fgfr1/2 then activates submesothelial Wnt2a to converge on β-catenin signaling that, in turn, promotes mesenchymal cell proliferation that is important for organ growth^14^ (Fig. 2b). Mesodermal β-catenin additionally promotes Fgf10+ PSMC progenitor amplification but plays no role in their differentiation to SMCs. However, proper endothelial cell differentiation is contingent on β-catenin signaling, indicating its necessity for the formation of multiple mesenchymal lineages via paired-like homeodomain transcription factor 2 (PITX2)-dependent mechanisms^15^.

Wnt7b is expressed throughout the airway epithelium by the start of the pseudoglandular stage, with the greatest expression localized to the distal airway, specifically the lung bud tips^35,36^ (Fig. 2b). Wnt7b loss-of-function mice retain proximodistal patterning despite their smaller lung architecture and incomplete cartilaginous ring formation^35,37^. Furthermore, autocrine Wnt7b signaling governs epithelial proliferation, in part, by Bmp4-dependent mechanisms^37^.

Epithelial Wnt signals delivered to the overlying mesenchyme also play an instrumental role in this stage of lung development. Endodermal-derived Wnt7b promotes lung mesenchymal proliferation and pulmonary vasculature growth in this stage^35,37^ (Fig. 2b), as its inactivation results in mouse lung hypoplasia, weakened vascular smooth muscle integrity, and death within minutes postnatally due to respiratory failure^35^. Wnt7b also provides a paracrine signal to the adjacent mesenchyme to promote Pdgfrβ+ pulmonary SMC proliferation and commitment to vascular SMCs^38^, which is mediated by extracellular matrix protein tenascin C^38^ (Fig. 2b).

As with the embryonic phase, tight regulation of canonical Wnt signaling activity is also important in the pseudoglandular stage. Notum, a deacylase that removes Wnt lipid modifications and therefore inhibits Wnt secretion, is critical for tracheal development^39^. Notum knockout mice exhibit tracheal stenosis, reduction in cartilaginous rings, and trachealis muscle by E16.5^39^. Notum is regulated by Wntless (Wls), a cargo protein involved in trafficking lipid-modified Wnt from the Golgi to the cell surface. Epithelial deletion of Wls also impairs tracheal development, branching morphogenesis, and proximo-distal patterning as early as E12.5^39,40^. Further, in cultured lung explants, canonical Wnt signaling inhibitor dickkopf1 (Dkk1) inhibits lung tree branching and mesenchymal differentiation by decreasing distal mesenchymal Wnt2a, Pdgfrα, fibronectin, and alpha-smooth muscle actin expression^20^.

Inhibition of canonical Wnt signaling in the pseudoglandular stage is also achieved by activation of non-canonical Wnt signaling. Epithelial expression of the Cadherin EGF LAG seven-pass G-type receptor 1 (Celsr1) and PCP protein 2 (Vangl2) proteins involved with PCP axis promote branching morphogenesis, as mice with mutations in either of these proteins display misshapen lungs and fewer, more narrow airway lumens^41^. In addition, transcription factor Gata binding factor 6 (Gata6) activates non-canonical receptor Frizzled (Fzd2), which inhibits canonical Wnt signaling to constrain bronchioalveolar stem cell expansion during development^42^. The loss of Gata6 in Sftpc+ lung epithelium leads to dilated airways and neonatal death^42^.

### Canalicular/saccular stages (E16.5-P4)

While dispensable for early proximo-distal patterning, canonical Wnt7b ligand promotes distal Aquaporin5+ ATI lung epithelial cell differentiation during the canalicular and saccular stages^35^. These findings stand in contrast to those found by another group identifying normal, preserved cell differentiation from Wnt7b^−/−^ mouse lungs^37^. Others report that β-catenin in Sftpc-expressing lung endodermal progenitor cells is necessary for the formation of terminal alveolar saccules, as its loss results in a proximalized lung with enlarged bronchial tubes as early as E13.5^43,44^. Ectopic Dkk1 expression phenocopies these findings, likely through activation of Fgf10-Fgfr2 signaling^44^. During this stage, β-catenin is sufficient but not necessary for Sftpc+ lung endodermal progenitor cell proliferation^45^. Constitutive activation of β-catenin decreases differentiation of distal pulmonary cell types and instead promotes ectopic expression of gut and intestinal cell lineages^45^. This is accomplished by β-catenin binding to the *N-myc*, *Bmp4*, and *Fgf* promoters^44^ (Fig. 2c).

### Alveolarization stage (P4–P21)

Although studies have well characterized the mechanisms underpinning several early stages of lung development and the involvement of Wnt signaling, this remains poorly understood in the alveolarization stage. Early studies by Mucenski et al. demonstrate that a constitutively active form of β-catenin results in Forkhead boxa2 (Foxa2) inhibition, thereby contributing to epithelial cell dysplasia and goblet cell hyperplasia^46^. More recently, a study found a wave of Wnt signaling emerges in the late sacculation/early alveologenesis stage, marked by the expansion of Wnt-dependent AXIN2+ alveolar type II (ATII) cells that contribute to the budding alveolus^47^ (Fig. 2d). In contrast, low Wnt signaling skews the cellular fate toward an alveolar type I (ATI)-like lineage^47^. These results not only indicate the critical role of β-catenin, but also suggest that its tight spatiotemporal regulation is important for proper respiratory epithelial differentiation.

### Prospective/discussion of Wnt signaling in lung development

While all of these studies have proven instrumental in allowing us to define the role of Wnt signaling throughout the varying stages of lung development, much remains to be learned. It is apparent that the phases of lung development are interdependent on each other, rendering it difficult to interpret the biology that is specifically responsible for a given phase in isolation from other phases of development. It is critical that we begin to further dissect the dynamic nature of niche interactions during distinct phases of development. As such, development of novel tools to achieve this level of granularity and isolation will yield more nuanced insight of not only Wnt signaling, but other signaling cascades, in lung developmental biology. In addition, the advent of in vitro organoid systems has and will continue to allow for us to better understand human airway and lung development specifically. At the molecular level, much remains to be understood about the dynamic receptor–ligand interactions. The seminal work of Szenker-Ravi et al. and Lebensohn et al. raises the question: how does Rspo2 mechanistically potentiate Wnt signaling in the absence of LGR receptors^4,30^? Under what circumstances is R-spondin interaction with its cognate LGR required for distinct phases of development?

## Submucosal gland biology

Submucosal glands (SMGs) are tubuloacinar structures of the submucosal region of the airway that are contiguous with the surface airway epithelium (SAE) by connecting ducts and play a critical role in mucous secretions that facilitate MCC, a function that is vital for host defense. SMGs contain several cell types including myoepithelial cells (MECs), ciliated cells, secretory cells, and basal cells. It was recently observed that SMGs also harbor glandular progenitors and that these cell types act as a reserve stem cell population to facilitate proximal airway regeneration following severe injury^48–51^.

In humans, SMGs localize throughout the trachea and bronchi of the proximal airway. However, in diseases such as cystic fibrosis, SMGs have been reported to extend more distally into the bronchioles of humans in addition to being more hyperplastic and hypertrophic^52^. In contrast, mice have primitive SMGs that are restricted to only the most proximal tip of the trachea. As such, ferrets and pigs have recently emerged as a more tractable model system to study SMG biology, as their SMGs develop postnatally and localize throughout the proximal airway and are therefore more similar to what is observed in humans^48,51^.

Early studies in ferret airways offered insight into *Lef1* localization at the invaginating tips of the SMG buds in early development^53^. Using human, ferret, and mouse model systems, Lymphoid enhancer-binding factor 1 (LEF1) was then shown to be necessary but not sufficient for SMG morphogenesis^52^ despite no reportedly appreciable changes in β-catenin nuclear localization^54^. Sox2 and Sox17 each function to suppress Wnt/β-catenin-mediated activation of the *Lef1* promoter^55–58^, indicating that these signaling axes are key regulators of SMG morphogenesis.

**Fig. 3:**
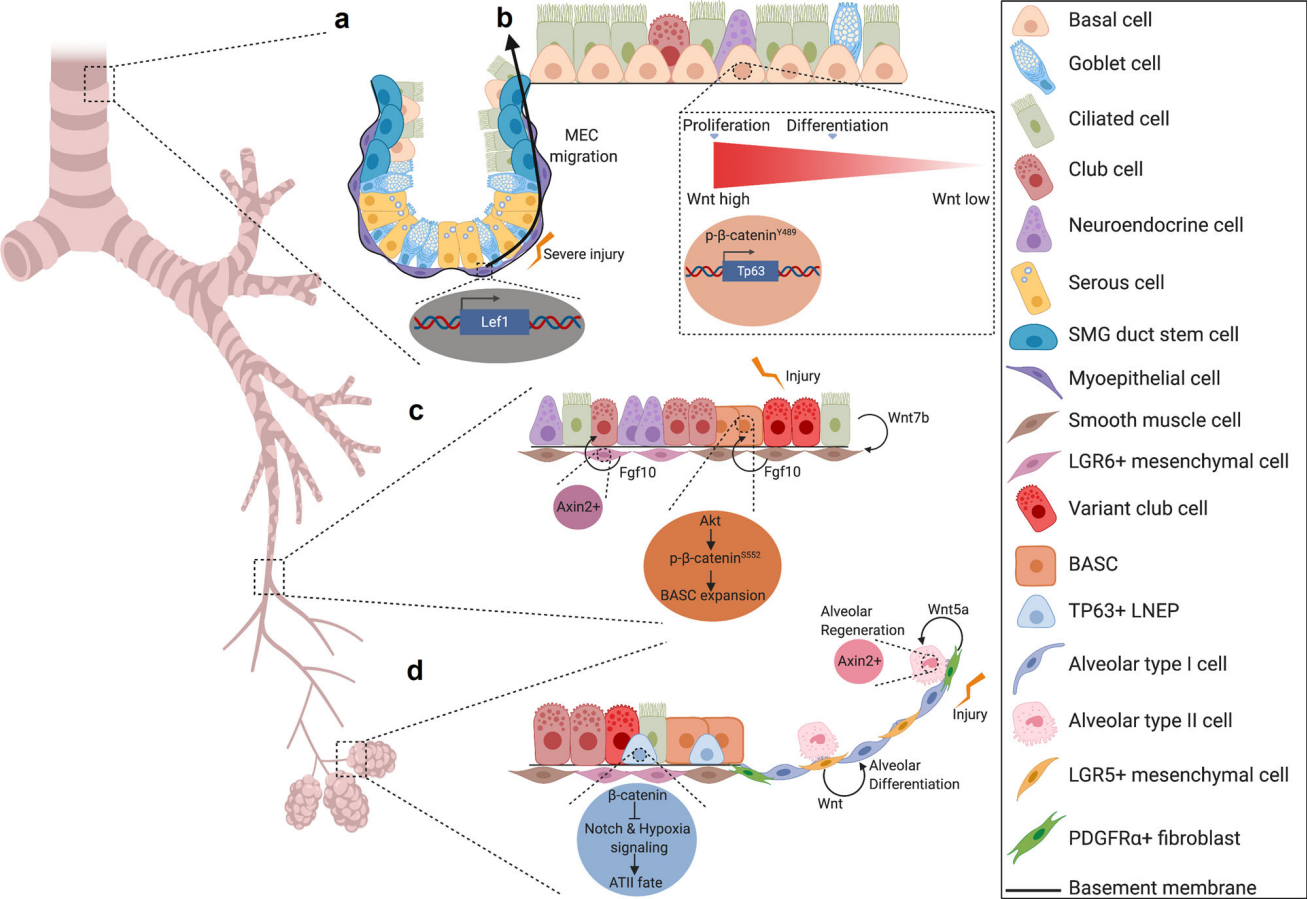
Wnt signaling plays pivotal roles in submucosal gland, proximal airway, bronchiolar, and distal lung regeneration. **a** Following severe injury, *Lef1* transcription within MECs of the SMGs promotes their migration to the surface airway epithelium and their subsequent ability to give rise to the differentiated cell types of the proximal airway^48,51^. **b** In the proximal airway ABSCs employ high levels of Wnt signaling to facilitate proliferation and medium levels of Wnt signaling to promote differentiation to the ciliated cell fate. At the molecular level, this appears to be accomplished by nuclear localization of p-β-catenin^Y489^ ^65^. Further, *Tp63* is regulated by upstream Wnt signaling to promote basal cell stemness, thereby inhibiting differentiation to ciliated cells^66^. **c** In the bronchioles after injury, ciliated cells produce Wnt7b ligand that signals to airway smooth muscle cells (SMCs). SMCs then secrete Fgf10 ligand to promote an Akt/p-β-catenin^S552^ signaling cascade. These events then, in turn, promote BASC expansion^76,77^. Another study identified that Wnt-responsive (Axin2+) Lgr6+ mesenchymal cells are a key source of Fgf10 ligand to promote bronchiolar repair^81^. **d** At the bronchioalveolar duct junction, LNEPs employ β-catenin signaling to inhibit Notch and hypoxia signaling that is permissive for formation of an ATII-like cell fate^80^. Further, Lgr5+ mesenchymal cells secrete Wnt ligands that promote alveolar differentiation^81^. In the alveolus, Pdgfrα+ fibroblasts are also a key source of Wnt5a to signal to Axin2+ ATII cells to drive alveolar regeneration^83^.

Wnt signaling is dynamically activated in SMGs following proximal airway injury concomitant with the proliferative phase of repair^59^. Further, Wnt3a is sufficient to promote glandular progenitor proliferation. Wnt-active cells reside near label-retaining cells of the SMG and were thought to be a regulatory component of the stem cell niche for repair^59^. This was later confirmed in a follow-up study that illustrated the capacity of MECs to migrate to the SAE and contribute to the repair process by giving rise to several differentiated cell types of the airway^48,51^. Further, *Lef1* overexpression is sufficient to induce cell movement and migration programs that promotes MEC commitment to differentiated lineages of the proximal airway^48^ (Fig. 3a).

## Proximal airway homeostasis and regeneration

The proximal airway harbors a pseudostratified epithelium that is comprised of basal cells, mucus-secreting goblet and secretory (club) cells, and ciliated cells. Under the majority of circumstances, the basal cell is the resident adult stem cell responsible for giving rise to differentiated progeny. However, because the trachea of mice has a slow turnover rate of approximately one cell every 7–11 days, injury repair models are often employed to understand the cellular dynamics of stem cell fate decisions^60^.

The development of an understanding for how Wnt signaling modulates proximal airway homeostasis and regeneration remains in its infancy. It is known that basal cell β-catenin signaling is dynamically activated and necessary for the proliferative phase in murine injury repair models^59,61–63^. In addition, as described above, Wnt signals facilitate MEC contribution to proximal airway regeneration following severe injury^48^. At the cellular level, a recent group identified that a subset of Pdgfrα+ cells in the intercartilaginous zone are transiently, dynamically activated to secrete Wnt ligand that signals to the airway epithelium to promote its proliferation^64^. During differentiation, β-catenin activity is increased in air–liquid interface (ALI) cultures, a culturing system used to model airway epithelial remodeling, and enhances specification for the ciliated cell lineage^61^. In vivo, this phase is also driven by Wnt secretion from K5+ ABSCs that are necessary for differentiation to the ciliated cell fate^64^.

While Wnt signaling promotes ciliated cell differentiation, this is not true at all doses of pathway activation^65^. In contrast, high levels of Wnt signaling drive ABSC hyperproliferation concomitant with a phosphorylated form of nuclear β-catenin at Y489 (p-β-catenin^Y489^) and abolishment of differentiation to the ciliated cell fate^65^ (Fig. 3b). Haas et al. demonstrated that high Wnt signaling triggers transcription of *Tp63*, which then acts as a master regulator of basal cell stemness^66^. A recently identified novel Wnt signaling inhibitor can promote restoration of Wnt-induced dysregulation of proximal airway homeostasis via p-β-catenin^Y489^/TP63-related mechanisms^65^ (Fig. 3b), further implicating the importance of the pathway in proximal airway homeostasis.

## Distal lung homeostasis and regeneration

As the trachea branches and forms the distal lung, there are appreciable changes in the cell types that act as stem or progenitor cells in homeostasis and regeneration that are spatially defined. In the mouse bronchiole, Scgb1a1+ Scgb3a2+ club cells, Scgb1a1− Upka3+ club cells, and Cgrp+ neuroendocrine cells can all act as stem/progenitor cells in either homeostatic or regenerative conditions^67–69^. The bronchoalveolar duct junction (BADJ) houses the Scgb1a1+ Sftpc+ Sca1+ bronchoalveolar stem cell (BASC)^70,71^. Further, Sftpc+ ATII cells in the alveolar region undergo self-renewal and give rise to ATI cells^72^. Most recently, a family of stem/progenitor cells referred to as the lineage-negative epithelial progenitors (LNEPs) have been characterized as quiescent at homeostasis but are mobilized under injurious conditions to regenerate the lungs^73,74^. Although the intricate complexity of the identity of these stem cell progenitors continues to be an area of current investigation, Wnt signaling plays critical roles in several of these regional compartments in regeneration.

From a cell biology perspective, several of the aforementioned identified progenitor populations or non-stem supporting niche cells employ Wnt signaling in bronchiolar, bronchioalveolar, or alveolar regeneration. Although an early study reported that β-catenin in club cells is dispensable for bronchiolar epithelial repair^75^, several subsequent findings indicate a highly important and intricately regulated role for this pathway at the cellular level in repair.

In the bronchioles and BADJ following naphthalene injury, ciliated cells induce Wnt7b expression that signals to the PSMCs to induce *Fgf10* expression^76,77^ (Fig. 3c). Mesenchymal Fgf10 then induces Ak strain transforming (AKT)-mediated phosphorylation of β-catenin at S552 to promote BASC expansion and subsequent epithelial regeneration^78^ (Fig. 3c). Constitutive activation of β-catenin increases bronchiolar stem cell expansion and attenuates differentiation^79^. β-catenin stabilization also skews sex-determining region Y-box 2 (Sox2)+ LNEPs toward an ATII-like rather than K5+ cell fate by inhibiting Notch and hypoxia signaling following influenza infection^80^ (Fig. 3d). More recently, a study also identified that bronchiolar Lgr6+ mesenchyme is a Wnt-responsive cell population that secretes Fgf10 to signal to promote epithelial club cell differentiation^81^ (Fig. 3c).

In contrast, Wnt production from the alveolar Lgr5+ mesenchyme facilitates alveolar differentiation^81^ (Fig. 3d). A subset of Pdgfrα+ alveolar fibroblasts are Wnt-responsive (AXIN2+) signal to adjacent to ATII cells to promote their self-renewal and differentiation to ATI cells^82^. Interestingly, Pdgfrα+ alveolar fibroblasts also serve as key source of Wnt5a that signals to Axin2+ ATII cells to facilitate their self-renewal during homeostasis^83^, together suggesting the potential dual function of Wnt production and Wnt responsiveness of a single-cell (Fig. 3d). In the context of injury, however, ATII cells become Axin2+^84^ and induce autocrine Wnt signaling to promote progenitor expansion^83^. A recent study also demonstrated that neutrophil transmigration facilitates epithelial regeneration by inducing β-catenin signaling in neighboring ATII cells^85^.

At the molecular level, transcription factors and co-activators within the nucleus also play pivotal roles in modulation of bronchiolar and alveolar progenitor differentiation. In the bronchioles, interaction between β-catenin and P300 facilitates mucus cell formation^86^. Further, β-catenin interaction with either c-AMP element response binding protein or P300 inhibits generation of ciliated cells^86^. In the alveolus, a Wnt5a/Pyruvate kinase C signaling axis also activates the interaction between P300 and β-catenin, which is necessary for ATII differentiation to ATI cells^87^. Transcription factor Gata6 also prevents excessive BASC expansion by suppressing canonical Wnt signaling in response to injury^42^.

An important contribution to the field of whole lung regeneration was made in 2019 with the work of Mori et al., who employed conditional blastocyst complementation approaches in mice^88^. These injected cells compete for a specific niche to subsequently undergo the developmental program within the recipient host. Mori et al. identified that wild-type donor cells injected at the blastocyst stage of *Ctnnb1* null mice were capable of giving rise to functionally mature lungs with mice living into adulthood. These efforts have collectively carved new strategies for whole lung regeneration in vivo.

### Prospective/discussion of Wnt signaling in SMG biology, airway, and lung regeneration

The recent identification of a novel reserve stem cell population holds promising implications for disease therapeutics. However, much remains to be learned about the molecular and cellular cues that trigger MEC migration following severe injury. In addition, it will be important to dissect the functional and cellular heterogeneity of MECs in the SMGs and how they may or may not differentially contribute to airway regeneration and/or disease progression. Although it is clear that Wnt signaling plays a pivotal role in SMG morphogenesis and contributes to repair, closer analyses at single-cell resolution will further refine the granularity of our understanding of its biology.

While these initial findings are important for the field, much remains to be explored. In particular, we need to better dissect the spatiotemporal relationship between the diverse cell types of the airway as it relates to Wnt signaling. Previous work has illustrated the critical importance of mesenchymal-epithelial cell crosstalk for mediating airway regeneration^64,89^. As such, these efforts should be expanded to the realm of Wnt/β-catenin signaling given its appreciated importance. Specifically, these studies have elucidated the structural, cellular, and molecular complexity and heterogeneity of the intercartilaginous zone niche that warrants further investigation. Future studies should seek to further identify and characterize the cell types that comprise this niche, as they will inherently inform our understanding of the mechanisms governing proximal airway regeneration. Further, although the use of in vitro ALI culturing systems has allowed for significant biological understanding of Wnt signaling in airway biology, this platform has inherent limitations. Differences in culture media and timing of treatments have the possibility to yield seemingly conflicting and contradictory results. Last, the creation of transgenic mouse model tools that can uniquely isolate and dissect the role of this pathway in proliferation versus differentiation in vivo, two distinct but co-variate processes, would greatly advance not only the Wnt field forward, but the field of airway biology as a whole.

## In vitro directed differentiation from iPSCs and ESCs and implementation in organoid models

Reliable, efficient differentiation of human PSCs and ESCs along airway and lung lineages has garnered the attention of several groups in recent years. These efforts have far-reaching implications for regenerative medicine, disease modeling, and forging opportunities for disease gene correction. These efforts gained prominence after Gouon-Evans et al. demonstrated the efficient enrichment of definitive endoderm from mouse embryonic stem cells (mESCs) via Activin A, a Nodal-like TGFβ family member known to induce an endodermal fate^90^. The use of Activin A has also been shown to drive definitive endoderm in human ESCs, mirroring developmental cascades observed in murine models^91^.

**Fig. 4:**
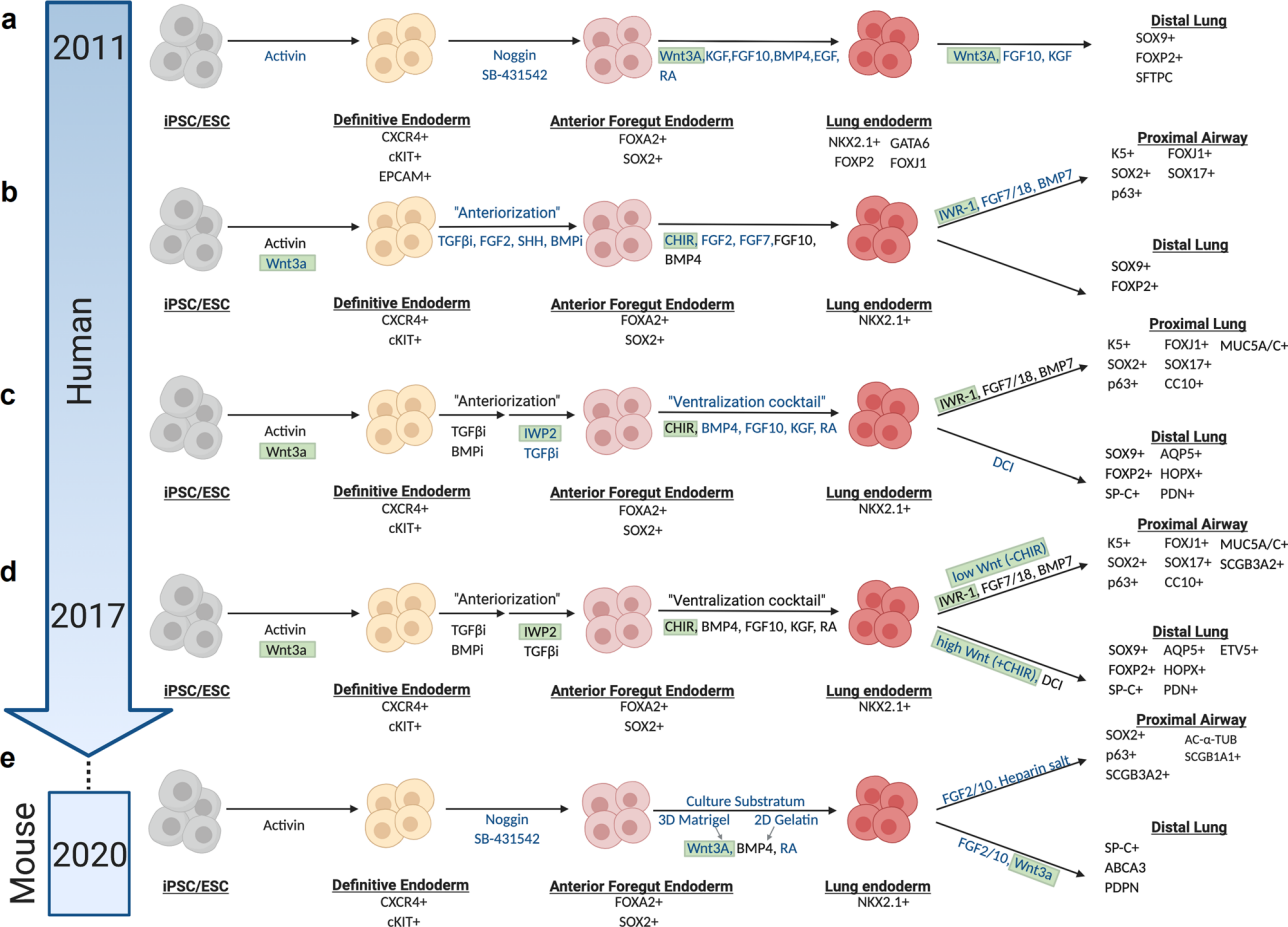
Directed differentiation of iPSCs to lung lineages utilizes perturbations in Wnt signaling at various stages. **a** Primary report to lung-directed differentiation utilized Activin to induce definitive endoderm specification. Use of Noggin and SB431542, a bone morphogenetic protein (BMP) and transforming growth factor beta (TGFβ) family signaling inhibitor, respectively, in Activin A-induced definitive endoderm drives anterior foregut endoderm (AFE) specification^92^. Further differentiation of the AFE would utilize a WKFBE factor cocktail (WNT3a, keratinocyte growth factor (KGF), fibroblast growth factor (FGF) 10, BMP4, epidermal growth factor (EGF)) alongside retinoic acid (RA) to attain lung endoderm progenitors (NKX2.1+)^92^. Consistent with idea that activated Wnt signaling promotes distal lung phenotype, treatment of lung endoderm progenitors with Wnt3a, FGF10, and KGF promotes a distal phenotype^92^. **b** Subsequent studies introduced Wnt3a, alongside Activin, to generate definitive endoderm. GSK3β inhibitor CHIR, alongside FGFs and BMPs, facilitates generation of NKX2.1+ lung endoderm from anterior foregut endoderm. Tankyrase inhibition via treatment with IWR-1 skewed cells toward a more proximal fate^95,99^. New additions to differentiation protocols are indicated in blue while Wnt-specific biology is highlighted in green. **c** Subsequent efforts utilized among other factors in a “ventralization” cocktail that allowed for more efficient derivation of NKX2.1+ lung endoderm from anterior foregut endoderm^96^. New additions to differentiation protocols are indicated in blue while Wnt-specific biology is highlighted in green. **d** Between 2014–2017, refined strategies emerged that indicate the importance of the level of Wnt modulation by the absence or addition of CHIR to skew cell fates toward a more proximal or distal fate from NKX2.1+ lung endoderm^98,100,101^. New additions to differentiation protocols are indicated in blue while Wnt-specific biology is highlighted in green. **e** Most recently, using mouse PSCs, Wnt3a was removed from the generative of definitive endoderm and instead utilized in a “distalization media” to skew Nkx2.1+ lung endodermal progenitors to a more distal lung epithelial fate^104–106^. New additions to differentiation protocols are indicated in blue while Wnt-specific biology is highlighted in green.

While the first reports of definitive endoderm derivation focused on midgut and posterior foregut endoderm specification^90,91^, the first report of lung-directed differentiation was described in 2011. Green et al. first illustrated accurate specification of FOXA2+ SOX2+ AFE via inhibition of BMP and TGFβ signaling in Activin A-induced definitive endoderm^92^ (Fig. 4a). Further differentiation of the AFE to NKX2.1+ lung endoderm progenitors was achieved through use of a WKFBE factor cocktail (WNT3a, KGF, FGF10, BMP4, EGF) alongside retinoic acid^92^ (Fig. 4a). Comparative analyses of mouse ESC- and iPSC-derived definitive endoderm have demonstrated that both display similar gene expression and global transcriptome profiles^93,94^.

Subsequent groups introduced modifications to the directed differentiation of iPSC/ESC described above. Mou et al. introduced a transient 1-day activation of canonical Wnt signaling in addition to the use of Activin A to generate definitive endoderm (Fig. 4b)^95^. Similar to prior reports, TGFβ inhibition was maintained to induce anteriorization (FOXA2+ SOX2+) of this lineage. Wnt activation via GSK3β inhibition was identified as necessary for BMP4-dependent generation of NKX2.1-expressing lung endoderm progenitors (Fig. 4b)^90,91,95^. These data are suggestive of a concise, tightly regulated gene expression patterning required to promote lung endodermal progenitor formation. Subsequent Wnt inhibition using tankyrase inhibitor IWR-1 alongside Bmp7/Fgf7/18 signaling perturbations skews the lineage toward a proximal airway phenotype, while Wnt activation favors distal phenotypes (Fig. 4b)^95^. While the study provided an avenue to proximal airway lineage formation, it did not indicate generation of functionally mature proximal and distal lung cell types.

To address this concern, Wong et al. optimized the stepwise differentiation of iPSCs-derived lung endoderm into proximal lineage-specific epithelial cells through dynamic additions of various growth factors targeting major developmental pathways including Wnt signaling^96^. Consistent with prior reports, Wnt3a alongside Activin A are important for the generation of definitive endoderm progenitors (CXCR4+ CKIT+) (Fig. 4c)^95^. Importantly, subsequent differentiation into AFE is a Wnt-independent process and instead dependent on FGF and SHH stimulation. Downstream proximal specification (K5+ P63+) additionally requires low BMP signaling^96^.

To contrast Fgf/Shh-mediated AFE induction (Fig. 4a), Huang et al. then demonstrated dual BMP/TGFB inhibition sequentially followed by Wnt/Tgfβ inhibition generates increased Nkx2.1+ AFE cells (Fig. 4c). Further, a “ventralization cocktail”, similar to that reported by Green et al., with the omission of EGF, comprised of GSK3β inhibitor CHIR99021 (hereafter, CHIR), Bmp4, Fgf10, KGF and RA together was used to induce the fraction of lung progenitors^92,97^ (Fig. 4c). Removal of Wnt agonist CHIR from the ventralization cocktail decreases the number of NKX2.1+ cells, highlighting its functionally necessary, non-redundant role in lung endoderm specification^97,98^ (Fig. 4c). Thus far, Wnt signaling has been implicated in definitive endoderm formation^95,96,99^, its inhibition for efficient AFE induction^97,98^, and its activation for lung endoderm formation^95^ (Fig. 4c).

Recapitulating the complex temporal and regional specificity of several signaling pathway activities in lung development has contributed to stunted progress in allowing for appropriate proximo-distal lung patterning from human PSCs. To address this, McCauley et al. generated NKX2.1+ lung epithelial progenitors from human PSCs using previously published, well-established protocols^98,100,101^. They later use cyclical Wnt signaling modulation via treatment with GSK3β inhibitor CHIR to preferentially skew distal lung patterning over proximal airway cell fates, similar to the patterning methodology described by Mou et al.^95,101^ (Fig. 4d).

Recent work has built on these efforts more recently by elucidating the minimum factors sufficient for functional ATII cell specification from lung endodermal progenitors, one of which is CHIR^102^. Interestingly, full ATII maturation necessitates transient Wnt signaling downregulation by removal and subsequent addback of CHIR^102^. These findings are consistent with prior work indicating a brief wave of low-level Wnt signaling in the latter stages of fetal distal lung development^47^. Most recently, Hurley et al. reported a more refined timeline indicating that CHIR removal from the media should be done for 4 days (starting day 17 of their protocol) and subsequently added back to promote cellular proliferation and subsequent generation of ATII cells^103^. In addition, the same group also utilized knowledge gained from Nkx2.1+ murine lung epithelial progenitors in vivo to efficiently generate proximalized or distalized airway epithelial cells^104^. Inspired by prior studies, Wnt3a was removed from the generation of the definitive endoderm and instead was included in a “distalization” media on sorted NKX2.1+ reporter cells^104–106^ (Fig. 4e).

All of these in vitro directed differentiation efforts have laid the groundwork for several follow-up foundational studies that have generated multi-ciliated cells^107,108^ and reproducible iPSC/PSC-derived lung organoids^109–111^. Dye et al. first described the use of these directed differentiation protocols to generate 3D organoid cultures^110^. More recently, Chen et al. further demonstrated their ability to undergo branching morphogenesis and proximo-distal specification^112^. Both groups were, however, consistent with their use of Wnt activation to promote ventralization of the AFE^110,112^. Several groups have additionally employed the use of human pluripotent stem cell-derived lung organoids, which uniquely contain both endoderm and mesodermal tissues, to model lung diseases such as pulmonary fibrosis and cystic fibrosis^101,102,113^ and to ultimately use them as a base for therapeutic drug screens^114,115^.

### Prospective/discussion of Wnt signaling in iPSC differentiation

Through all stages of lung development, Wnt signaling, or its absence, plays a pivotal role in directing cellular differentiation. While Wnt signaling appears to be dispensable for definitive endoderm formation and is absent during anterior foregut patterning, its subsequent activation directs lung endoderm formation and distal lung specification. Many of these Wnt activation studies involve treatment with CHIR and it is important to note, however, that the effects of CHIR cannot be attributed only to Wnt signaling alone and may be perhaps due to Wnt/β-catenin-independent, GSK3β-dependent biology. Further, some studies have indicated variability in directed differentiation efficiencies between iPSC and ESC lines^96,98,101^. Taken together, these barriers in iPSC and ESC lung specification ultimately suggest a potential need for patient-specific differentiation protocols within the sphere of precision medicine in order to bypass cell–cell variability.

## Disease pathogenesis

In light of its highly complex involvement across several normal biological processes, it follows that dysregulation of the Wnt/β-catenin signaling cascade plays a similarly prevalent role in the pathogenesis of lung disease processes, including carcinogenesis, idiopathic pulmonary fibrosis (IPF), bronchopulmonary dysplasia (BPD), and chronic obstructive pulmonary disease (COPD) among others.

### Lung cancer

Lung cancer kills more people than breast, colon, and prostate cancers combined, with an overall 5-year survival rate that remains 18%. Although mutations in the Wnt/β-catenin signaling pathway are common in malignancies in several tissue types, including colon and intestine, they remain rare in lung cancer^116–119^. However, early studies using β-catenin gain-of-function animals crossed with Nkx2.1 tissue-specific Cre recombinase mice developed tracheal and bronchial polyps that contain an undifferentiated epithelium that surrounds a mesenchymal core^120^, implicating a role for the pathway in carcinogenesis. Subsequently, a myriad of other studies has demonstrated its key role in lung tumor burden progression as well as chemoresistance, as described below.

### Non-small cell lung cancer (NSCLC)

NSCLC is the most common histologically defined subtype of lung cancer, accounting for 85–90% of all lung cancers, and for which the most is known with respect to Wnt signaling. While activated Wnt/β-catenin signaling alone cannot drive tumorigenesis of the bronchiolar epithelium, its activation in the setting of *KrasG12D* mutations accelerates disease progression^121^. Further, *KrasG12D*;*p53*^*fl/fl*^;*Prkci* (KPI) lung adenocarcinoma (LADC) mouse model tumors arise from Tm4sf1 + Axin2+ ATII cells whereas *KrasG12D*;*p53*^*fl/fl*^ (KP) tumors arise from BASCs^122^. In addition, KPI tumors are reliant on higher levels of Wnt/β-catenin signaling for their expansion, a signature that is also reflected in human patient tumors^122^.

NSCLC tumors with constitutively active β-catenin induce a more distal embryonic lung phenotype that also represses E-cadherin, consistent with a role for Wnt/β-catenin signaling in EMT-like phenotypic processes that may facilitate metastasis to distant sites^62,121^. Indeed, a subset of LADCs uniquely display Wnt target gene transcriptional profiles that are associated with and drive metastatic potential to brain and bone^123^. Specifically, TCF1, TCF4, LEF1, and HOXB9 are key transcription factors that mediate cancer cell metastatic behavior^123^.

While many efforts have mechanistically dissected the molecular biology of Wnt signaling within the Wnt-responsive cancer cells, it is also important to recognize the source of Wnt ligand that triggers these downstream signaling and cellular events. Recent work has identified the presence of Wnt-producing (Porcupine+) epithelial cells that are closely apposed to Wnt-responding (LGR5+) epithelial cells in LADC^124^. Human premalignant lesions and squamous lung cancer samples also display increased stromal and epithelial Porcupine as well as nuclear p-β-catenin^Y489 65^, further elucidating the Wnt signaling niche networks that are targetable with small molecule inhibitors.

### Small cell lung cancer (SCLC)

SCLC is a rare, yet aggressive disease characterized by tumors with neuroendocrine features, expressing markers such as Chromogranin A, Synaptophysin, UCHL1, ASCL1, and NEUROD1. Although initially responsive to chemotherapy, resistant disease rapidly emerges, accounting for its high mortality rate. Tracheal and bronchial polyps with constitutive activation of β-catenin contain higher levels of neuroendocrine marker Uchl1^120^. The Wnt pathway is also implicated in the biology underpinning chemoresistance, as negative regulators of the pathway, *APC* and *CHD8*, were mutated in one-third of relapsed patients relative to their primary lesion samples prior to chemotherapy treatment^125^. In vitro APC knockdown studies conferred activated Wnt signaling and subsequent chemoresistance in SCLC cell lines^125^. Future studies in both NSCLC and SCLC should investigate the cellular heterogeneity of the transcriptional profiles of Wnt production and responsiveness in the context of chemoresistance biology.

### Idiopathic pulmonary fibrosis (IPF)

IPF is a rare, progressive disease marked by chronic lung scarring that results in poor lung function and for which there is currently no therapy to halt or reverse the fibrosis. The role of Wnt signaling in IPF remains convoluted, although studies have observed nuclear β-catenin in bronchiolar lesions, alveolar structures, and fibrotic foci^126^, specifically p-β-catenin^Y489 127^. Subsequent studies further elucidated a pathogenic role of the pathway in the disease process that is associated with poor prognosis^128^. Specifically, Wnt/β-catenin signaling is activated in humans with IPF and mouse models of the disease in both the mesenchyme as well as epithelium^129–132^. Further, administration of various Wnt signaling inhibitors attenuates the fibrotic phenotype both in vitro and in vivo^129,130,133^.

Wnt target gene Wnt-inducible signaling protein 1 is responsible, in part, for the fibrotic phenotype and decreased survival observed in animal models^129^. Further, LRP5 promotes β-catenin driven fibrosis by, in part, increasing TGFβ abundance^128^. LRP5 also acts to regulate the differentiation of alveolar macrophages and promote persistence of fibrosis^134^. In addition, the TGFβ pathway activates canonical Wnt signaling in human fibroblasts^130^ as well as induces extracellular vesicle secretion and subsequent release of Wnt5a ligand^135^. Together, these events trigger fibroblast proliferation and the fibrotic response. These findings also suggest multiple nodes of crosstalk between Wnt and TGFβ signaling cascades in IPF that warrants further investigation.

At the single-cell level, Wnt-producing cells are distinct from Wnt-responsive cells in humans with IPF^136^, suggesting the presence of an intricate epithelial-mesenchymal niche that parallels work identifying its functionality in normal injury repair of the distal lung^83^. Once internalized, the Wnt-responsive ATII cell secretes IL1β, which may then act as a profibrotic cytokine^137^.

In contrast, some have purported a protective role for β-catenin in the alveolar epithelium in bleomycin mouse models of fibrosis by promoting wound healing^138^. Taken together, these findings suggest complex, multi-faceted roles for Wnt/β-catenin signaling in IPF prevention, pathogenesis, persistence, and resolution.

### Other lung diseases

Bronchopulmonary dysplasia (BPD) is a rare pediatric lung disease characterized by abnormal distal lung development, declined lung function, increased inflammation, and increased fibrosis. BPD is often seen in premature infants or neonates that have received mechanical ventilation. In vitro and in vivo models of BPD employing hyperoxia injuries demonstrate reliance on mesenchymal Wnt5a on disease phenotypes^139^. BPD also shares a common downstream activated, phosphorylated form of β-catenin at Y489 that is also observed in IPF^127,140^, though much remains to be explored in this disease context. COPD also displays perturbed non-canonical Wnt signaling biology, as pulmonary fibroblasts secrete Wnt5a to inhibit alveolar canonical Wnt/β-catenin signaling, thereby preventing epithelial repair^141^. Recent work has also begun to think about the role of Wnt signaling in the context of aging as well. Lehmann et al. reported that aged ATII cells exhibit increase senescence that is driven by activation of Wnt/β-catenin signaling and is associated with profibrotic changes^142^.

### Prospective/discussion of Wnt signaling in disease biology

The work of Nabhan et al. has paved the way for understanding the role of single-cell niches within distal lung regeneration^83^. The success of this lies, in part, because its generated understanding of the Wnt-producing (Wnt-expressing, Porcupine-positive) cells and the Wnt-responsive (Axin2+) cells via single-cell sequencing studies. This approach should be adopted in the context of disease processes, as it could hold particular promise with disease processes with known intermediates such as the malignant transformation underpinning lung squamous cell carcinoma. There also exists a plethora of outstanding questions largely underexplored within the sphere of aging. For example, how do stem cell niches throughout the airway and lung change with aging, both at the cellular and molecular levels? And how, if at all, do the alterations in Wnt signaling that occur in aging relate to distinct pathophysiology of disease processes?

## Summary

Taken together, it is clear that Wnt signaling plays a major role in lung development, lung repair and regeneration, and the progression of many lung diseases. The rapid technological advances in the fields of molecular and cellular biology are greatly facilitating the study of Wnt signaling in lung biology. Advancing our knowledge on the exact mechanisms of Wnt signaling in the lung will allow for the development of more Wnt pathway targeted therapies that will hopefully lead to a therapeutic benefit for patients with lung diseases.
